# Medical Properties of the Scullcap, (Scutellaria Laterifolia)

**Published:** 1851-11

**Authors:** 


### MATERIA MEDICA AND THERAPEUTICS.

*Medical Properties of the Scullcap, (Scutellaria laterifolia.)*—Dr. Hunton, of Hyde Park, Vt., speaks very highly of this indigenous plant, as a remedy in hysteria, chorea, and other nervous diseases. The S. *galericulata*, or *European scullcap*, which also grows wild in this country, possesses, he says, similar medicinal properties and is less disagreeable to the taste. He also considers the Scutellariæ to be among our most valuable vegetable tonics.—*Abridged from N. J. Medical Reporter, Oct.* 1851.

